# Supplementary material for: Adaptation to Environmental Variability Shapes Dormancy in Daphnia
Source: bioRxiv. 2026 May 7:2026.05.06.723256. Preprint. [Version 1] doi: 10.64898/2026.05.06.723256 (PMC13174508; doi:10.64898/2026.05.06.723256)
Supplement: Supplement 1 [file NIHPP2026.05.06.723256v1-supplement-1.pdf]

bioRxiv preprint doi: https://doi.org/10.64898/2026.05.06.723256; this version posted May 7, 2026. The copyright holder for this preprint (which was not certified by peer review) is the author/funder, who has granted bioRxiv a license to display the preprint in perpetuity. It is made available under aCC-BY 4.0 International license.

STable 1: Coordinates of each Pond sampled

Pond	Coordinates (NW)
D8	50.642483, -2.091652
DBunk	50.64259 -2.09237
DCat	50.64245, -2.09121
Dcreech1	50.649302, -2.103066
Dcreech2	50.649698, -2.103930
DIris	50.644786, -2.083525
DLily	50.642690, -2.090075
DMountie	50.645270, -2.087337
DNorden	50.644915, -2.082837
DOak	50.64321, -2.09183
DOily	50.64321, -2.09183
DRail	50.655259, -2.093745
DRamps	50.643076, -2.090195
DTiny	50.642978, -2.092374
Birdhut	53.19086, -0.632353
P25	53.295671, -3.008023
PVet	53.290366, -3.021249
Cafe	52.060338, 0.7088274
Warbler	52.0606234, 0.7089288
Shallow	52.060193, 0.7090227
Melford	52.0587460, 0.7091847
XXX3	52.055213, 0.820963
P6	52.082746, 0.828459
XXX5	52.099159, 0.947914
P30	54.047405, -1.425774

bioRxiv preprint doi: https://doi.org/10.64898/2026.05.06.723256; this version posted May 7, 2026. The copyright holder for this preprint (which was not certified by peer review) is the author/funder, who has granted bioRxiv a license to display the preprint in perpetuity. It is made available under aCC-BY 4.0 International license.

Stav1	54.062929, -1.436836
Stav2	54.059490,-1.437444
Stav3	54.063553, -1.440872
PottCarr1	53.496057, -1.113718
PottCarr2	53.496096, -1.111111
PottCarr3	53.495725, -1.112190
P17	51.346052, -0.931059
Islands	51.339417, -0.908203
Canal	51.1079946, -0.5350191
P19	51.111720, -0.524786
Elvis	53.1973519, -0.63267486
PSnake	N/A

bioRxiv preprint doi: https://doi.org/10.64898/2026.05.06.723256; this version posted May 7, 2026. The copyright holder for this preprint (which was not certified by peer review) is the author/funder, who has granted bioRxiv a license to display the preprint in perpetuity. It is made available under aCC-BY 4.0 International license.

STable 2: COI reference panel and accession numbers

Species	COI/Mitochondrial accession number
Daphnia obtusa FS6	>CM028013.1
Daphnia obtusa NA1	>AY380443.1
Daphnia obtusa NA2	>AY380446.1
Daphnia pulex KAP4	>AF117817.1
Daphnian pulicaria	>AF489523.1
Daphnia obtusa EU	Elvis3
Daphnia pulex EU	mtdna_D8_119
Daphnia pulicaria EU	Pulicaria_Pond22_53
Daphnia arenata	>FJ427493.1
Daphnia pulex	>KF993372.1
Daphnia galeata	>NC_034297.1
Daphnia pileata	>AY380453.1
Daphnia ambigua	>AF523699.1
Daphnia magna	>KP296147.1
Daphnia carinata	>KP721459.1
Daphnia parvula	>HM883991.1
Daphnia mitsukuri	>MK434272.1
Daphnia melanica	>FJ427495.1
Daphnia magna	>PP824112.1
Daphnia catawba	>AY380454.1
Daphnia longispina	>MW201533.1
Daphnia sinensis	>PV089802.1
simocephalus vetulus	>OQ713637.1
